# A Patient With Epilepsy, Ganglioglioma, and Oligodendroglioma With Anaplastic Foci in the Same Left Frontoparietal Lesion: A Case Report

**DOI:** 10.7759/cureus.31323

**Published:** 2022-11-10

**Authors:** Keren Magaly Aguilar-Hidalgo, José Alfonso Alvarez-Castro, José Omar Santellán-Hernández, Ana Laura Calderón-Garcidueñas, Gerardo Romero-Luna, Gonzalo Monjarás-Romo, Jorge Alejandro Torres-Ríos, Sonia Iliana Mejía-Pérez

**Affiliations:** 1 Neurosurgical Oncology Department, Instituto Nacional de Neurología y Neurocirugía “Manuel Velasco Suárez”, Mexico City, MEX; 2 Neuropathology Department, Instituto Nacional de Neurología y Neurocirugía “Manuel Velasco Suárez”, Mexico City, MEX; 3 Neurosurgery Department, Instituto Nacional de Neurología y Neurocirugía, Mexico City, MEX; 4 Radiosurgery Department, Instituto Nacional de Neurología y Neurocirugía “Manuel Velasco Suárez”, Mexico City, MEX; 5 Neurosurgery Department, Instituto Nacional de Neurología y Neurocirugía “Manuel Velasco Suárez”, Mexico City, MEX

**Keywords:** awake patient surgery, neurosurgery, brain tumor, oligodendroglioma, ganglioglioma

## Abstract

Gangliogliomas are central nervous system (CNS) tumors with a neuronal and glial component considered grade 1 according to the World Health Organization (WHO) classification. On the other hand, oligodendrogliomas are diffuse infiltrating gliomas (CNS WHO grade 2 or 3) characterized by both an isocitrate dehydrogenase mutation and 1p/19q co-deletion. There have been some cases with the coexistence of these two tumors. Here, we present the case of a low-growing left frontoparietal brain tumor with a definite diagnosis of ganglioglioma (CNS WHO grade 1) and oligodendroglioma (CNS WHO grade 2) with areas of anaplastic oligodendroglioma (CNS WHO grade 3) in a patient with long-standing epilepsy.

## Introduction

Gangliogliomas are grade 1 tumors (World Health Organization (WHO) classification), constituting 0.4-1.4% of central nervous system (CNS) tumors. These tumors have a special predilection for the temporal and frontal lobes, where they are associated with epileptic seizures, and are resistant to medical treatment. These tumors include neurons and neoplastic glial components [1,2].

Oligodendroglioma is a diffusely infiltrating, slow-growing glioma, categorized as grade 2 or 3 according to the WHO classification. Although the most frequent location is in the frontal lobe, the temporal lobe is also frequently involved. These tumors have a microscopic appearance of “fried egg” cells, with a profile characterized by mutations in the *isocitrate dehydrogenase* (*IDH*) gene and 1p/19q co-deletion [3,4]. Oligodendroglial neoplasms are considered glial tumors; however, they may have neuronal and neurocytic differentiation capacity [5].

Malignant change rarely occurs in gangliogliomas, but when it does occur, it involves the astrocytic glial component [6]. Yamashita et al. reported an oligodendroglial and astrocytic component in a patient with an initial ganglioglioma [2]. In this manuscript, we present the case of a patient with a ganglioglioma who subsequently developed an oligodendroglioma with anaplastic areas.

Seizures are the presenting symptom in approximately two-thirds of patients with oligodendrogliomas [7]. Patients with this type of neoplasm may also present with headaches due to increased intracranial pressure and focal neurological deficit, depending on the location of the tumor. With advanced imaging becoming more widely available for symptom screening, incidental diagnoses are more frequently reported, accounting for 10% of cases in one study [5].

## Case presentation

A 34-year-old man with a smoking history since the age of 17 and social alcoholism reported that his maternal grandmother had suffered from ovarian carcinoma and hemangioblastoma. He began his current condition in 2019, with tonic-clonic seizures in the right hand for one minute. At that time, a brain computed tomography (CT) scan showed a tumor in the left frontal lobe, for which surgery was proposed, and diazepam was prescribed to control the seizures.

**Figure 1:**
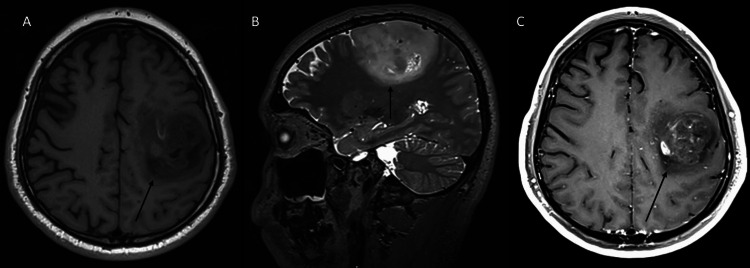
Preoperative cranial MRI (May 2021). (A) T1-weighted cranial MRI, axial section; (B) T2-weighted cranial MRI, sagittal section; (C) with-contrast cranial MRI, axial section. An intra-axial lesion seen in the frontoparietal region, with involvement of the precentral and postcentral gyri; a heterogeneous lesion is identified in all sequences, with some hyperintense areas in T1; discrete and heterogeneous enhancement is observed with contrast medium. MRI: magnetic resonance imaging

However, due to the severe acute respiratory syndrome coronavirus 2 pandemic, contact between the institute and the patient was lost from 2019 to 2021, during which time the seizures continued, with approximately five episodes per day, lasting one minute per episode. In May 2021, given the persistence of seizures, the patient returned to the hospital. A with-contrast brain magnetic resonance imaging (MRI) scan was requested (Figure 1). Seizures were treated with clonazepam one tablet every 12 hours, phenytoin one tablet every eight hours, and lacosamide 50 mg every 12 hours.

**Figure 2:**
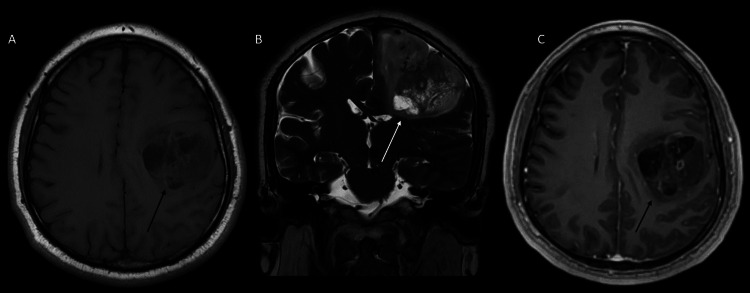
Brain MRI (April 2022). (A) T1-weighted, axial section; (B) T2-weighted, coronal section; (C) contrasted BRAVO, axial section. Left frontoparietal intra-axial lesion, solid heterogeneous with hyperintense areas in T1, is surrounded by slight perilesional edema, affecting the superior frontal gyrus, middle frontal gyrus, pre-postcentral gyrus, conditions mass effect with caudal compression of the adjacent convolutions, as well as the frontal recess and medialization of the uncus. Intravenous contrast shows heterogeneous and irregular enhancement. Compared to the previous study, it presents greater extension and mass effect. MRI: magnetic resonance imaging

**Figure 3:**
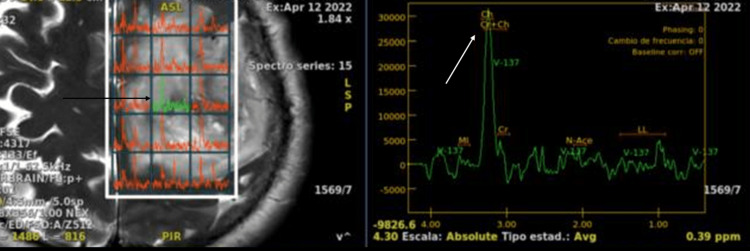
Brain MRI, spectroscopy (April 2022). In spectroscopy, at the lesion site, there is a ratio of Ch/Cr 4.51, Na/Cr:1.23. There are peaks of MI, lipids, and lactate. MRI: magnetic resonance imaging

In April 2022, he again suffered a convulsive crisis in the right hand, along with asthenia, adynamia, progressive weakness of the right side of the body, and language alterations. Surgical resection of the lesion was scheduled. On physical examination, the patient was alert, oriented, and cooperative, but presented motor dysphasic language. He named and repeated appropriately. Right facial paralysis and normal tone and trophism were observed. Strength evaluation showed right upper extremity strength of 0/5, right lower extremity 1/5, and left hemibody 5/5. Clonus in the right arm and leg were documented. The sensory examination was normal, with Karnofsky of 70 points. A new MRI was requested (Figures 2, 3).

Due to the location of the tumor, which was found in eloquent motor and language areas, such as the motor area, Broca’s area, upper longitudinal tract, and arcuate fasciculus, it was decided to protocolize surgery for tumor resection in an awake patient. Tests were carried out by the psychology and anesthetic teams.

In July 2022, asleep-awake-asleep surgery was performed, with intraoperative, ultrasound-guided, neuronavigation. Intravenous fluorescein 10% (2 mL) was used before durotomy to delimit tissue where the blood-brain barrier had been broken. Penfield bipolar cortical stimulation was also used to delimit the eloquent zones of movement and speech. Once a safe area for resection was identified, surgery was performed. Eloquent areas in the tumor tissue were identified and it was decided to preserve them. The patient had a good postoperative recovery. He was discharged four days after surgery, with an improvement in dysphasia, and without any visible improvement in the motor component.

**Figure 4:**
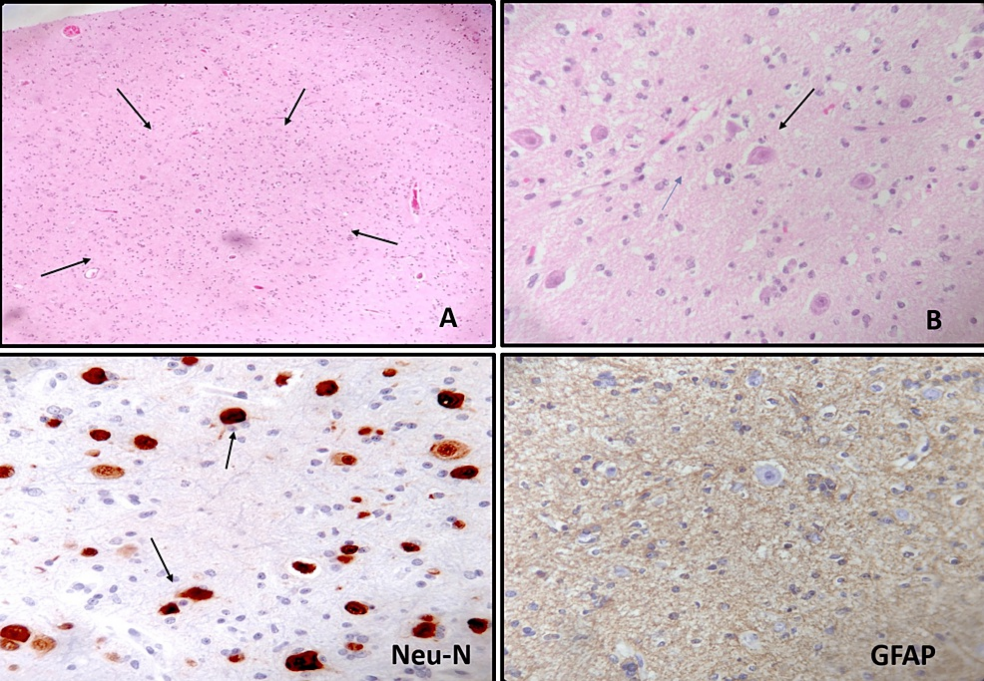
Ganglioglioma histology. The cerebral cortex showing an alteration in the structure of its layers, with an irregular accumulation of large ganglion-like neurons (A: 100× and B: 400×, arrows), Neu-N+, and an astrocytic component GFAP+.

**Figure 5:**
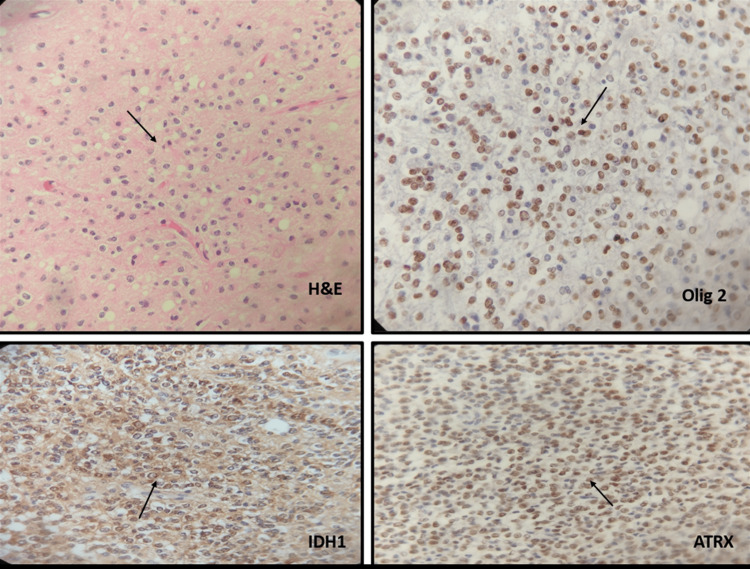
Oligodendroglioma histology. Oligodendroglioma with “fried egg look” of individual cells, olig 2+, IDH1+, and ATRX retention.

The histopathological study of the tissue showed several tumor areas with different cell types described below. In the cortex, the structure of the layers was altered by the presence of clusters of ganglion-like neurons, confirmed by Neu-N positivity (Figure 4). An increase in the surrounding capillaries and peripheral glial component, GFAP+, without atypia, was also identified. Toward the depth of the cortex, an area with cells in a mosaic pattern was observed, individually, with a fried egg look, accompanied by long and delicate capillaries. Neoplastic cells stained with immunohistochemical staining for Olig-2 showed *IDH1 *mutation and ATRX retention (Figure 5).

**Figure 6:**
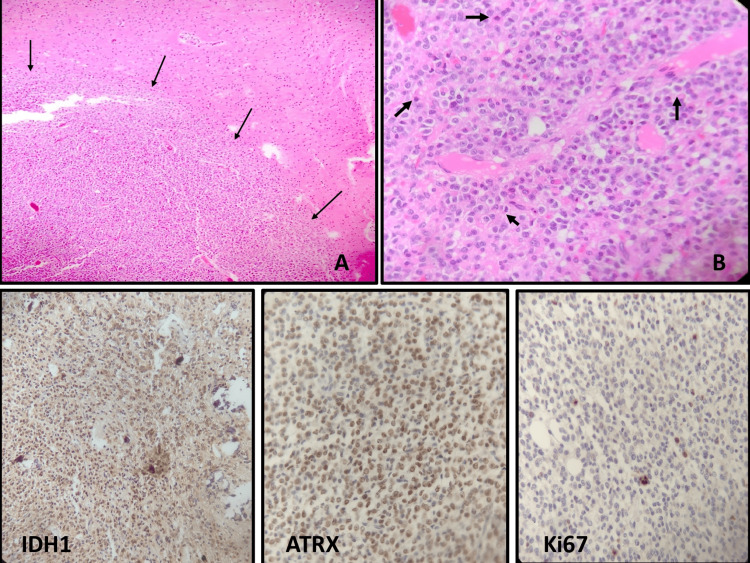
Anaplastic oligodendroglioma histology. Anaplastic oligodendroglioma area with increased cellularity (A), numerous mitoses (B, arrows), and *IDH1 *positivity, ATRX retention, and high proliferation index (Ki67+).

Finally, an area with increased cell density was also observed, which preserved the morphology of oligodendroglioma, but presented nuclear hyperchromasia and mitosis, Ki67 nuclear positivity, *IDH1 *mutation, and retention of ATRX (Figure 6).

## Discussion

The distinction between ganglioglioma and oligodendroglioma is critical because the WHO tumor grade is different, and, therefore, patients’ treatment strategies and prognoses also differ. Ganglioglioma is a grade 1 tumor that can be cured by complete resection. However, oligodendroglioma requires adjuvant therapy, such as radiotherapy, and frequent follow-up. Generally, when the report shows a CD34-negative and IDH1+ tumor by immunohistochemistry (IHC), it excludes the diagnosis of ganglioglioma; however, there are reports demonstrating an *IDH*-positive mutation in ganglioglioma-like areas [3].

OLIG2 is a transcription factor that plays an important role in the specification of oligodendrocyte precursors and is useful to show the oligodendroglial nature. If an oligodendroglial tumor is suspected, a 1p/19q codeletion search should be done because it constitutes the hallmark alteration in oligodendroglioma in at least 80% of the cases [8].

In a report by Yamashita et al. [2] in 2011, oligodendrogliomas with ganglioglioma-like maturation showed that both neuronal and glial components had the 1p/19q codeletion. CD34- immunoreactive cells as an important marker should be screened to consider dysplastic glial cells in gangliogliomas. Dysembryoplastic neuroepithelial tumor could be a differential diagnosis but differs from gangliogliomas and oligoastrocytoma in negative staining for GFAP [2,9].

In the WHO brain tumor update, a rare tumor known as diffuse glioneuronal tumor with oligodendroglioma-like features and nuclear clusters (DGONC) is included [1]. DGONC has a distinctive methylation profile different from previously recognized CNS tumor molecular groups. Histologically, it has clear cell features with high cellularity, isomorphic, and round nuclei, with oligodendroglioma-like perinuclear halos, vascular proliferation, and neuropil-like islands. The tumor cells do not express astrocytic markers such as GFAP, but they are positive for neuronal markers, synaptophysin, and NeuN, as well as the oligodendrocyte marker OLIG2 and strongly positive for MAP2. Monosomy 14 is not found exclusively in DGONC, although it is close to 100% in this type of tumor [10,11].

Some authors proposed hypotheses about the development of collision tumors, in which different histological types such as astrocytoma, glioblastoma, or ganglioglioma with meningioma were found. Theories have been proposed about gangliocytoma undergoing anaplastic changes that may give rise to anaplastic oligodendroglioma. Thus, the preexisting gangliocytoma could have transformed into a ganglioglioma with an oligodendroglioma component [12].

Oligodendrogliomas-gangliomas must be differentiated from central neurocytomas (CNs). These usually arise in the anterior half of the lateral ventricles and are commonly intraventricular tumors. The age of onset is in the second or third decade of life (mean age of 29 years), with no predilection on gender. Similar to gangliomas, they are mostly circumscribed and calcified tumors. Histologically, these CNs have small round nuclei with perinuclear halos. However, focal necrosis, endothelial proliferation, and mitotic activity are not common in CNs [13].

Residual tumor masses, frontal tumor location, and histological atypia or anaplasia, as found in our case with an anaplastic oligodendroglioma component, are associated with a greater risk of recurrence or malignant progression [14,15]. Therefore, due to the anaplastic component, the patient was referred to the neuro-oncology service for further treatment.

## Conclusions

We report the case of a patient with a four-year history of seizures, with initial imaging documentation of a low-grade neoplasm, probably related to the ganglioglioma found in the resection, in which, in addition to the ganglioglioma, an oligodendroglioma with anaplastic areas was found (collision tumor). The profile of both components was defined by IHC, and given the location of the lesion, functional surgery was performed with good postoperative evolution.
